# Comparative Growth Performance and Intestinal Morphological Development Between Liangshan Yanying Chicken and Arbor Acres Chicken During the Brooding Stage

**DOI:** 10.3390/ani16060991

**Published:** 2026-03-22

**Authors:** Ziheng Sun, Tao Li, Chao Chen, Chengpeng Wu, Ruyun Zhuo, Dan Wang, Qianwen Deng, Chaoyun Yang, Jing Wang, Heng Yang, Zengwen Huang

**Affiliations:** 1 College of Animal Science, Xichang University, Xichang 615000, China; xcdaxue@xcc.edu.cn (Z.S.); xcc20210230@xcc.edu.cn (T.L.); xcc20220311@xcc.edu.cn (C.C.); zy1074681078@outlook.com (C.W.); zhuoruyun@foxmail.com (R.Z.); 13541413320@163.com (D.W.); dqianwen0303@163.com (Q.D.); chaoyuny@yeah.net (C.Y.); 2 College of Tourism and Urban-Rural Planning, Xichang University, Xichang 615000, China; slnee.5703@163.com; 3 College of Veterinary Medicine, Southwest University, Rongchang, Chongqing 402460, China; yhuv2013@sina.com

**Keywords:** Liangshan Yanying chicken, AA broiler, growth performance, intestinal morphology, villus height, feed conversion ratio

## Abstract

The differences in growth performance and intestinal morphology between Liangshan Yanying Chicken (YYJ) and Arbor Acres (AA) broilers during the brooding stage, and to explore the effect of early intestinal development on growth. A total of 120 one-day-old male chicks of each breed were selected and randomly divided into three groups, reared under the same conditions for 28 days to measure body weight, feed intake, and intestinal morphological indices. The results showed that the body weight of AA broilers at 28 days of age was 3.24 times that of YYJ; the average daily gain and average daily feed intake from 1 to 28 days of age were 3.11 and 2.36 times those of YYJ, respectively, while the feed conversion ratio decreased by 24.10% (*p* < 0.01). Regarding intestinal morphology, the density of each intestinal segment in AA broilers remained significantly higher than that in YYJ. At 1 day of age, the villus height of the duodenum and ileum was 14.44% and 39.20% higher, respectively (*p* < 0.05 or *p* < 0.01), and the villus-to-crypt ratio in most intestinal segments at various ages was 78.27% to 91.05% higher (*p* < 0.01). Correlation analysis indicated that body weight at 14 and 28 days of age was significantly positively correlated with the average daily gain of each stage (*p* < 0.01), and significantly negatively correlated with the feed conversion ratio (*p* < 0.01). These findings indicate that the difference in early intestinal morphological development may constitute a key factor contributing to the growth limitation of local chicken breeds. This study provides a theoretical basis for the genetic improvement and nutritional regulation of YYJ.

## Simple Summary

This research compares the growth and gut development of Liangshan Yanying Chicken and AA Broilers during their first 28 days. The study aims to understand why local breeds grow slower by examining their intestinal structures. Findings show that AA Broilers grew over three times larger and used feed much more efficiently. This advantage is linked to their superior gut structure, featuring higher density and longer villi—tiny projections that absorb nutrients. These features provide a larger surface area for digestion and lower energy costs for maintenance. While Liangshan Yanying Chicken grows more slowly, its longer intestines reflect an adaptation to its native high-altitude environment. The study concludes that early gut development is a primary factor limiting the growth of local breeds. This is valuable to society as it supports the genetic improvement of Liangshan Yanying Chicken, helping to preserve local poultry resources and meet the demand for high-quality meat.

## 1. Introduction

In the contemporary poultry industrial system, growth performance and feed conversion efficiency represent the core indicators for evaluating the economic value of breeds [1]. Body weight (BW), average daily gain (ADG), and feed conversion ratio (FCR) constitute the fundamental framework for assessing broiler production performance, where ADG directly reflects the rate of weight gain and FCR quantifies the efficiency of feed conversion into body mass [2]. Simultaneously, the gastrointestinal tract serves as the critical organ for nutrient digestion and absorption, with its developmental status directly constraining growth rates and feed utilisation efficiency [3]. Research indicates that small intestinal development in broilers precedes that of body tissues post-hatching, and early morphological construction exerts a decisive influence on production performance throughout the entire growth cycle [4]. At the histological level, villus height (VH), crypt depth (CD), and the villus height to crypt depth ratio (V/C) are key parameters for evaluating small intestinal function: an increase in VH signifies an expanded absorptive surface area; CD reflects the turnover rate of epithelial cells; and the V/C value comprehensively characterises the functional state of the intestinal mucosa [5,6,7]. Therefore, elucidating the differences in intestinal morphological development between breeds is of great significance for resolving the differentiation in growth performance from a biological mechanism perspective.

China possesses abundant indigenous poultry genetic resources, and local chicken breeds are highly favoured in the market due to their strong adaptability and unique meat flavour [8]. In recent years, relevant studies have predominantly focused on genetic diversity conservation and production performance assessment [9]. In contrast, commercial broiler lines, such as the AA broiler, have achieved rapid growth and efficient feed conversion through long-term directional selection [10]. Comparative studies show that indigenous chicken breeds in southern China generally exhibit higher crude protein content, lower shear force values, and smaller muscle fibre diameters, with meat tenderness and flavour quality surpassing those of fast-growing commercial broilers [11]. However, local chicken breeds often underperform compared to commercial lines in terms of early growth rate and feed efficiency, and the physiological underpinnings of these divergent growth patterns require further investigation. As a representative indigenous breed adapted to high-altitude environments, YYJ provides an ideal model for studying the trade-off between growth performance and environmental adaptation. YYJ is an indigenous dual-purpose (meat and egg) breed from Meigu County, Liangshan Prefecture, Sichuan Province, and is included in the National Catalogue of Livestock and Poultry Genetic Resources. Its core production areas are located in alpine regions at altitudes of 1800–2000 m, where long-term natural selection has endowed the breed with robust stress resistance and the ability to thrive on coarse fodder. This breed is noted for its tender meat and rich flavour, possessing both nutritional value and medicinal health functions, making it a high-quality indigenous poultry resource with significant developmental potential.

In recent years, while research on YYJ germplasm resources has made some progress, studies systematically exploring its early digestive tract morphological development and its correlation with growth performance remain relatively scarce. Considering that the intestine is the core organ for nutrient digestion and absorption, and its morphological development directly affects growth rate and feed utilization efficiency, we hypothesize that the significant differences in growth performance between YYJ and AA broilers may be closely related to their differences in early intestinal morphological development. Therefore, this study selected YYJ and AA broilers as controls. By systematically measuring growth indicators (body weight, daily gain, feed conversion ratio) and small intestinal histological parameters (VH, CD, V/C) during the early developmental stages (days 1, 14, and 28), this study aims to reveal the differences in early growth patterns and intestinal development between local and commercial breeds, thereby elucidating the histological basis of growth performance differences.

## 2. Materials and Methods

All experimental procedures were conducted in strict accordance with the Guidelines for the Care and Use of Laboratory Animals formulated by the Animal Ethics Committee of Xichang University. The experimental protocol received formal approval from the Animal Ethics Committee of Xichang University (Approval No.: xcc2022003). The principles of Reduction and Refinement were strictly applied to minimize both the animal count and procedural discomfort.

### 2.1. Experimental Animals and Grouping

Hatching eggs of YYJ were sourced from the Xinyi Ecological Breeding and Planting Professional Cooperative in Yuexi County, Liangshan Prefecture, China, while AA broiler eggs were obtained from the Chia Tai Group (Parent Stock Farm in Suining, China). A total of 200 eggs from each breed were disinfected, weighed, and numbered before undergoing simultaneous incubation in a “Fuhui” integrated incubator and hatcher (Wuhan Fuhui Co., Ltd., Wuhan, China). The incubation conditions involved a temperature of 37.8 °C and a relative humidity of 60% from days 1 to 18 (with egg turning every 2 h), followed by 37.2 °C and 70% humidity from days 19 to 21. Candling was performed on embryonic days 7, 11, and 19 to remove infertile eggs and dead embryos. The hatching rate for the Liangshan Yanying Chicken was 82.5% (165 chicks from 200 eggs), while the hatching rate for the AA Broilers was 88.0% (176 chicks from 200 eggs). Following hatching, 120 healthy chicks from each breed were selected for rearing. For each breed, three replicate cages were established, with each cage containing 40 chicks, and additional chicks were reserved as backups. For performance traits (ADFI and FCR), the cage (replicate) was defined as the experimental unit (*n* = 3 per breed). For individual measurements (BW and intestinal morphology), the individual bird was the observational unit, with the cage effect accounted for in the statistical model.

### 2.2. Rearing and Management

The experimental period lasted 28 days, during which the birds were reared by group in intelligently temperature-controlled brooding incubators, with YYJ and AA broilers housed separately. All replicate cages for both breeds were housed in the same environmentally controlled room. The positions of the cages were interleaved to eliminate potential confounding effects between breed and micro-environmental factors. Before the trial, the incubators were spray-disinfected with 0.1% benzalkonium bromide. The ambient temperature was maintained at 33–36 °C for days 1–7 and reduced to 30–33 °C for days 8–14. From day 15 onwards, the temperature was gradually decreased by 1 °C per day until room temperature was reached. A lighting regimen of 23 h light and 1 h darkness was implemented throughout the study. Feeding occurred twice daily (08:00 and 20:00), with daily records kept for feed offered and residual feed per group. The chicks had ad libitum access to feed and water, and excreta removal and disinfection were performed daily. To ensure the uniqueness of experimental variables, no routine immunisation programmes were administered. Both breeds were fed the same air-dried pellet feed (starter complete feed for broilers, purchased from Kunming Bangyun Feed Co., Ltd., Kunming, China; dietary formulation is provided in Table S1).

### 2.3. Measured Indices and Methods

#### 2.3.1. Growth Performance Measurement

On days 1, 14, and 28, the individuals were weighed following a 12 h fast (with free access to water) [12]. Accurately record feed intake (calculated by feeding amount) for specific time periods (days 1–14, 15–28, and 1–28). The following parameters were calculated:

Average Daily Gain (ADG, g/d) = (Final Weight − Initial Weight)/Number of Days.

Average Daily Feed Intake (ADFI, g/d) = (Total Feed Offered − Residual Feed)/Number of Days.

Feed Conversion Ratio (FCR) = Total Feed Intake/Total Weight Gain.

#### 2.3.2. Intestinal Histomorphology Measurement

1. Relative Length, Relative Weight, and Density

On days 1, 14, and 28, after weighing and blood sampling, 10 chicks per group from each breed were randomly selected and euthanised via jugular vein exsanguination. The entire gastrointestinal tract was removed, and the duodenum, jejunum, ileum, and caecum were separated using the U-loop junction, yolk sac vestige, and ileocecal junction as anatomical landmarks. After removing the pancreas, mesentery, and adipose tissue, intestinal segments were temporarily immersed in sterile Petri dishes containing PBS. After expelling the contents, each segment was straightened until retraction ceased and measured with a scale (0.1 cm precision). Following gentle drying with absorbent paper, segments were weighed (0.01 g precision) to calculate relative weight, relative length, and density. Body length was defined as the distance from the tip of the beak to the base of the tail (uropygium), measured using a flexible ruler (0.1 cm precision) with the chick placed in a natural prone position, and all measurements were performed by the same operator to ensure consistency.

For histological observation, mid-sections (approximately 1.0–2.0 cm in length) of the duodenum, jejunum, and ileum were collected. These segments were immediately and gently flushed with cold sterile physiological saline to remove any residual digesta. Subsequently, the cleaned tissues were submerged in 4% paraformaldehyde for at least 24 h for histological fixation.

Relative Weight (%) = Intestinal segment weight/Liveweight

Relative Length = Intestinal segment length/Body length × 100%

Density (g/cm) = Intestinal segment weight/Intestinal segment length

2. Villus Height and Crypt Depth

Intestinal tissues of both breeds were fixed, dehydrated through graded ethanol, cleared in xylene, and embedded in paraffin. Sections (4 μm) were cut, mounted on slides, and stained with Hematoxylin and Eosin (H&E) [13]. Digital images were captured using a PANNORAMIC 250 scanner (3DHISTECH, Budapest, Hungary) (https://www.3dhistech.com). For each section, five complete villi and crypts were measured using Image-Pro Plus 6.0 (https://www.mediacy.com/imageproplus (4 June 2022)) (in μm) to calculate the villus height-to-crypt depth (V/C) ratio.

### 2.4. Statistical Analysis

Raw experimental data were standardised to calculate derivative indices such as ADG, FCR, and relative intestinal density. Independent-samples *t*-tests were employed to examine significant differences between the two breeds. For comparisons involving three or more groups, One-way Analysis of Variance (ANOVA) was utilised, with the significance level set at α = 0.05. Correlation analysis was performed using SPSS 24.0 software, calculating Pearson correlation coefficients to assess the linear relationship between body weight, growth rate, and intestinal histomorphological indices (VH, CD, V/C), thereby elucidating the correlation between intestinal development and growth performance. Statistical results are presented as “Mean ± Standard Deviation (Mean ± SD)”. Significant differences are denoted by different lowercase letters or “*” for *p* < 0.05, and by different uppercase letters or “**” for *p* < 0.01. Data visualisations were generated using GraphPad Prism 9.0 (https://www.graphPad.com) and R (4.5.2) language.

## 3. Results

### 3.1. Comparative Analysis of Egg Weight and Body Weight at Different Ages During the Brooding Stage Between Liangshan Yanying Chicken and AA Broilers

Body weight measurements (Table 1) revealed that the egg weight, as well as the body weights at days 1, 14, and 28, were significantly lower in YYJ than in AA broilers (*p* < 0.01). Specifically, the egg weight of AA broilers was 22.44% higher than that of YYJ (62.87 vs. 51.35 g). The 1-day-old body weight of AA broilers exceeded that of YYJ by 29.07% (44.71 vs. 34.64 g). This weight disparity widened further to 168.87% by day 14 (433.96 vs. 161.38 g). By day 28, the body weight of AA broilers reached 3.24 times that of YYJ (1279.56 vs. 394.49 g).

### 3.2. Comparative Analysis of Growth Performance at Different Ages During the Brooding Stage Between Liangshan Yanying Chicken and AA Broilers

**Figure 1:**
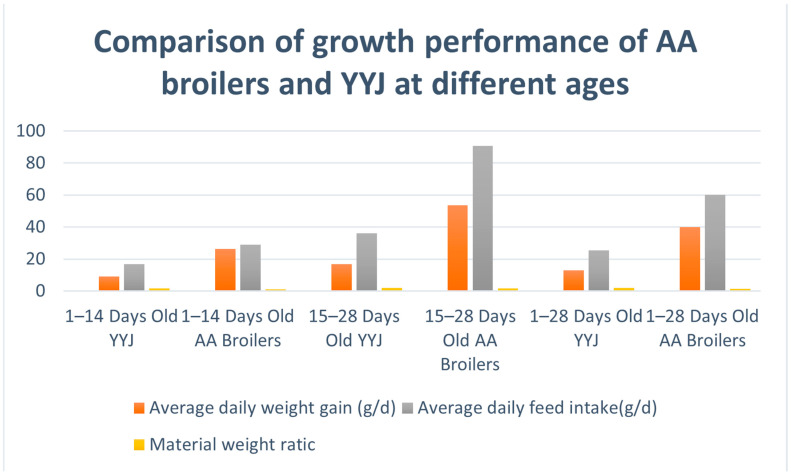
Comparison of growth performance between YYJ chickens and AA broilers. Note: YYJ denotes Liangshan Yanying chicken; AA broilers denotes Arbor Acres chicken.

Growth performance comparisons during the brooding stage (Table 2, Figure 1) revealed that the average daily gain (ADG) and average daily feed intake (ADFI) of AA broilers were significantly higher than those of YYJ across all stages (*p* < 0.01). From days 1 to 14, the ADG of AA broilers was 2.87 times that of YYJ (26.13 vs. 9.10 g/d), and the ADFI was 71.35% higher (28.92 vs. 16.88 g/d). Between days 15 and 28, the disparity in ADG expanded to 3.21 times (53.49 vs. 16.65 g/d), with the ADFI difference reaching 150.55% (90.56 vs. 36.14 g/d). Over the entire period (days 1–28), the ADG and ADFI of AA broilers were 3.11 and 2.36 times those of YYJ, respectively.

In terms of feed conversion efficiency, AA broilers exhibited significantly lower feed conversion ratios (FCR) than YYJ throughout all experimental stages (*p* < 0.01). Specifically, the FCR of AA broilers was 37.79% lower than that of YYJ from days 1 to 14 (1.07 vs. 1.72), 18.84% lower from days 15 to 28 (1.68 vs. 2.07), and 24.10% lower over the entire period (1.48 vs. 1.95).

### 3.3. Correlation Analysis of Egg Weight and Body Weight at Different Ages During the Brooding Stage Between Liangshan Yanying Chicken and AA Broilers

Pearson correlation analysis (Table 3) revealed a significant relationship between egg weight and early brooding body weight. In YYJ, egg weight exhibited a significantly positive correlation with 1-day-old body weight (r = 0.620, *p* < 0.01) and a significantly positive correlation with 14-day-old body weight (r = 0.405, *p* < 0.05). Additionally, significantly positive correlations were observed between 1-day-old and 14-day-old body weights (r = 0.416, *p* < 0.05), as well as between 14-day-old and 28-day-old body weights (r = 0.495, *p* < 0.05). For AA broilers, egg weight was significantly and positively correlated with 1-day-old body weight (r = 0.823, *p* < 0.01) and significantly positively correlated with 14-day-old body weight (r = 0.496, *p* < 0.05). In addition, a significantly positive correlation was identified between 1-day-old and 14-day-old body weights (r = 0.552, *p* < 0.01), while 14-day-old body weight showed a significantly positive correlation with 28-day-old body weight (r = 0.490, *p* < 0.05).

### 3.4. Comparative Analysis of Intestinal Morphology Between the Two Breeds During the Brooding Stage

#### 3.4.1. Macroscopic Intestinal Morphological Indices

The comparison of intestinal development indicators (Table 4) revealed significant differences in the macroscopic intestinal morphology between the two breeds, exhibiting an age-dependent variation pattern.

Relative Intestinal Length: At 1 day of age, the relative length of the duodenum in YYJ was significantly greater than that in AA broilers (26.94% vs. 24.34%, *p* < 0.01). By days 14 and 28, the relative lengths of all intestinal segments (duodenum, jejunum, and ileum) in YYJ were significantly greater than those in AA broilers (*p* < 0.01). Taking day 28 as an example, the relative lengths of the duodenum, jejunum, and ileum in YYJ were 2.08, 1.90, and 1.79 times those of AA broilers, respectively.

Relative Intestinal Weight: At days 1 and 14, the relative weights of all intestinal segments in YYJ were significantly lower than those in AA broilers (*p* < 0.01). Nevertheless, by day 28, a reversal occurred in the relative weights of the jejunum and ileum, which became significantly higher in YYJ than in AA broilers (jejunum: 1.04% vs. 0.84%, *p* < 0.01; ileum: 0.82% vs. 0.67%, *p* < 0.01), whereas the difference in the relative weight of the duodenum was not significant.

Intestinal Density: Across all observed ages, the density of each intestinal segment in YYJ remained consistently and significantly lower than that in AA broilers (*p* < 0.01). At 1 day of age, the densities of the duodenum, jejunum, and ileum in AA broilers were 33.33%, 50.00%, and 50.00% higher than those in YYJ, respectively. By day 28, these disparities further expanded to 75.00%, 58.33%, and 55.56%.

#### 3.4.2. Intestinal Microscopic Structure

The results of histological analysis are shown in Table 5. Villus Height (VH): At 1 day of age, the duodenal villus height of AA broilers was significantly higher than that of YYJ (270.16 vs. 236.08 μm, *p* < 0.05). With the exception of the jejunum at day 1, the VH across all intestinal segments and ages remained significantly greater in AA broilers compared to YYJ (*p* < 0.01). At 1 day of age, for instance, the VH of the duodenum, jejunum, and ileum in AA broilers exceeded those of YYJ by 14.44%, 6.92%, and 39.20%, respectively. By day 14, the disparity in jejunal VH expanded to 32.41% (89.30 vs. 67.45 μm).

Crypt Depth (CD): The jejunal crypt depth in AA broilers was consistently and significantly deeper than that in YYJ at all observed ages (days 1, 14, and 28; *p* < 0.01). At 28 days of age, AA broilers also demonstrated a significantly advantage in duodenal and ileal CD (*p* < 0.01), which were 56.38% and 16.85% higher than those of YYJ, respectively.

Villus Height to Crypt Depth Ratio (V/C): The V/C ratio serves as a critical indicator of integrated intestinal function. At day 1, the V/C values in all intestinal segments of AA broilers were significantly greater than those of YYJ (*p* < 0.01), with increases of 78.27%, 91.05%, and 90.24% in the duodenum, jejunum, and ileum, respectively. At day 14, only the duodenal V/C remained significantly higher than that of YYJ (143.43 vs. 110.40, *p* < 0.01). By day 28, the V/C values across all intestinal segments in AA broilers were again comprehensively and significantly superior to those in YYJ (*p* < 0.01).

## 4. Discussion

The comparative analysis reveals systemic and significantly differences in growth performance between YYJ and AA broilers during the brooding stage. The comprehensive advantages observed in AA broilers regarding body weight, growth rate, and feed conversion efficiency are fundamentally rooted in their highly diverged genetic backgrounds compared to indigenous breeds.

### 4.1. Influence of Genetic Background on Growth Disparities During the Brooding Period

Breed variation represents the primary driver for significant differences in body weight, average daily gain (ADG), and feed intake. As a modern commercial breed subjected to intense directional selection, the genetic core of the AA broiler is engineered for extremely rapid muscle deposition and growth. Conversely, the genome of YYJ, an indigenous breed, preserves a greater abundance of genetic variants associated with environmental adaptation and stress resistance [14,15]. Genomic research by Youm et al. [15] on Korean Long-tail chickens demonstrated that loci related to environmental adaptation in indigenous breeds have undergone strong positive selection, whereas loci directly linked to muscle growth rates lack enrichment. Similarly, studies on Wenchang chickens by Gu et al. [14] reached consistent conclusions. These divergent selection pressures at the genomic level fundamentally determine the differing growth potentials between indigenous and commercial lines.

These disparities manifest as early as the embryonic stage. The significantly higher egg weight of AA broilers provides a more substantial yolk nutrient reserve, resulting in a higher birth weight [16,17]. Wilson’s [18] classic review indicates that for every 1 g increase in egg weight, birth weight rises by approximately 0.5–0.7 g, with correlation coefficients typically ranging from 0.7 to 0.9. In this study, the correlation coefficient between egg weight and day-old body weight of AA chickens was 0.878 (*p* < 0.01), aligning closely with established biological laws. Notably, Wilson also observed that the influence of egg weight on post-hatch weight diminishes with age [4,18]. This observation is consistent with the findings of the present study where the correlation between egg weight and 28-day-old weight was not significant, suggesting that post-hatch feed intake and intestinal digestive capacity gradually supersede maternal nutrient reserves as the dominant factors limiting subsequent growth [19].

This initial weight advantage enhances the individual’s feed intake, creating a virtuous cycle of “high initial birth weight → strong feed intake → high daily weight gain,” enabling AA chickens to grow rapidly during the brooding period. van de Ven et al. [17] similarly found that chicks with superior initial developmental status exhibit stronger feeding drive and faster growth rates post-hatching. This feedback mechanism implies that the early weight gap between the two breeds amplifies as brooding progresses; indeed, the weight disparity expanded from 1.29-fold at day 1 to 3.24-fold at day 28 in this study, providing direct evidence of this mechanism in action.

### 4.2. Relationship Between Intestinal Morphological Development and Feed Conversion Efficiency

The significant differences in intestinal morphology between Liangshan Yanying Chicken and AA broilers likely contribute to their divergent FCR. While we lack direct digestive physiological data, the superior villus height and V/C ratio in AA broilers suggest an increased absorptive surface area, which is physiologically associated with enhanced nutrient utilization. Thus, these morphological variations serve as a key contributing factor to the observed differences in growth performance and feed efficiency. Following hatching in avians, the small intestine is one of the first organs to initiate rapid development, and its morphological and functional maturity directly constrains the efficiency of exogenous nutrient utilisation [4,20]. Nitsan et al. [20] noted that during the first week post-hatching, the relative growth rate of the small intestine far exceeds that of body tissues, a “gut-prioritised development” pattern that is particularly pronounced in fast-growing breeds.

At the microscopic level, AA broilers construct an intestinal absorptive barrier significantly superior to that of YYJ during early brooding. Higher villus height (VH) greatly expands the effective absorptive surface area of the intestinal mucosa, directly enhancing nutrient uptake efficiency [21,22]. Notably, at day 1, the ileal VH of AA broilers was 39.20% higher than that of YYJ, indicating that AA broilers possess a more mature mucosal structure immediately upon hatching. A large-scale cross-sectional study of 50 commercial broiler farms by Rysman et al. [23] provides robust validation—they found that duodenal VH and V/C ratios correlated significantly with ADG (r = 0.431 and r = 0.539, *p* < 0.05), while crypt depth (CD) correlated significantly with FCR (r = 0.389, *p* < 0.05), supporting a strong association between intestinal morphology and production performance under commercial conditions. However, the increased intestinal crypt depth in YYJ may also reflect accelerated epithelial cell turnover—a high-energy compensatory response to environmental or dietary stress. This compensatory process requires a significant amount of energy for tissue repair, which may increase “maintenance costs” and ultimately lead to a higher FCR in this breed.

Furthermore, the high V/C ratios observed in AA broilers across all intestinal segments imply higher epithelial cell maturity and lower endogenous energy expenditure for tissue turnover, thereby directing more nutrients toward body tissue deposition [24]. The V/C ratio is regarded as a more valuable integrated indicator than VH or CD alone because it simultaneously reflects “absorptive capacity” and “maintenance cost” [7,25]. In comparisons of broilers with differing growth rates, Gorenz et al. [25] found that individuals with high V/C values exhibited lower FCR, identifying V/C as a reliable marker for intestinal maturity. In this study, the V/C values of AA broilers at day 1 were 78.27–91.05% higher than those of YYJ. This superior microstructure, coupled with high feed intake, constitutes the physiological prerequisite for the extremely low FCR in AA broilers [26,27].

Nevertheless, at day 14, the V/C advantage in AA broilers was observed only in the duodenum, with no significant differences in the jejunum and ileum (Table 5). This transient attenuation may be attributed to the asynchronous development of different intestinal segments and the dynamic intestinal remodelling process during the second week post-hatch. Studies have shown that the duodenum develops preferentially over the jejunum and ileum as the first segment receiving digesta [28,29]. Furthermore, the period around day 14 coincides with the transition from endogenous to exclusive exogenous nutrition, during which intestinal epithelial cells undergo dramatic adjustments in proliferation and apoptosis rates, potentially causing transient morphological fluctuations [30,31]. This phase has been described as an “intestinal functional remodelling period”, where villus height and crypt depth may temporarily decline or stagnate to adapt to rapid changes in dietary composition [4]. Given the faster growth rate of AA broilers, their intestinal remodelling process may be more intense, temporarily masking the V/C advantage in the jejunum and ileum at day 14. By day 28, as intestinal function stabilises, the V/C advantage re-emerged across all segments, consistent with their lower overall FCR (1.48 vs. 1.95). This observation suggests that future studies should incorporate sampling points with higher temporal resolution (e.g., days 7 and 21) to more precisely delineate the dynamic trajectory of intestinal development [32,33].

### 4.3. Adaptive Strategies of Intestinal Development in the Two Breeds

This study further demonstrates a significant co-developmental pattern between growth performance and intestinal morphological indices, though this pattern differs markedly between the two breeds, reflecting distinct adaptive strategies.

The rapid growth of AA broilers benefits from efficient intestinal absorption, where high ADG and low FCR are tightly coupled with high VH, intestinal density, and V/C ratios. This superior morphology provides an expanded surface area and optimal mucosal status, facilitating effective nutrient conversion. The intestinal density of AA broilers was significantly higher than that of YYJ at all ages (*p* < 0.01), with the gap widening over time—reaching a 75.00% difference in the duodenum at day 28. Intestinal density reflects the tissue mass per unit length; high density implies a more developed villus layer, thicker muscularis, and richer vascular network, enabling efficient digestion within a relatively shorter tract [11,34]. These findings align with studies by Lee et al. [27] and Alagbe et al. [35]. Huang et al. [34] also observed that high-efficiency individuals achieved superior digestion via higher density and VH despite having shorter relative intestinal lengths.

The synergistic developmental pattern of YYJ reflects a compensatory adaptation strategy. YYJ compensates for its lower absorption efficiency by increasing the relative length and weight of its intestine. This morphological feature is consistent with the “resource allocation theory,” which states that slower-growing breeds tend to develop larger digestive tracts to maximize the utilization of roughage or low-energy feeds [36]. Although this study has not directly measured indicators such as digestive enzyme activity or intestinal absorption function, the above results are consistent with the observations on intestinal morphological adaptation of local chicken breeds in previous studies [37,38,39]. This structural remodelling can be regarded as a compensatory mechanism to offset lower metabolic levels or specific nutritional limitations. Further research combining physiological and molecular mechanisms is needed in the future to fully elucidate the functional significance of this morphological variation. At days 14 and 28, the relative lengths of all intestinal segments in YYJ were significantly greater than those in AA broilers (*p* < 0.01); at day 28, the duodenal, jejunal, and ileal lengths were 2.08, 1.90, and 1.79 times those of AA broilers, respectively. This “length-for-area” strategy is biologically significant: a longer intestine extends digesta transit time and increases the contact between digestive enzymes and nutrients, thereby improving overall nutrient extraction under conditions of lower unit-area efficiency [40,41,42]. Schmidt et al. [41] found a similar pattern when comparing modern broilers to unselected 1950s lines.

Interestingly, the relative intestinal weight of YYJ exhibited age-dependent dynamics. While lower than AA broilers at days 1 and 14, the relative weights of the jejunum and ileum in YYJ surpassed those of AA broilers by day 28. This reversal suggests an adaptive remodelling process in the late brooding stage. Gabella [42] noted that when the intestine faces increased functional loads, the intestinal wall undergoes smooth muscle hypertrophy and thickening. However, this delayed compensatory response contributes minimally to overall growth; the relative lag in intestinal development during the critical day 1–14 window establishes the foundation for the final weight divergence between the two breeds.

From an ecological perspective, the strategy of lengthening the intestine in YYJ does not constitute a “disadvantage”; rather, it may represent an adaptation to their native high-altitude environment (1800–2000 m). In such regions, where feed resources are limited and high in fibre, a longer intestine enables more thorough digestion and provides greater space for microbial fermentation, thereby facilitating energy extraction from coarse fodder [14,40,41]. Studies on migratory birds also suggest that plasticity in intestinal length is a common ecological strategy for coping with fluctuations in food availability [43,44]. However, when fed high-energy commercial diets, this adaptive advantage becomes a burden, as YYJ cannot match the rapid and efficient nutrient conversion achieved by AA broilers.

### 4.4. Limitations and Perspectives

This study has certain limitations regarding sample size and duration. Only 10 birds per breed per age were euthanised for intestinal measurements, which may not fully capture individual variation. The 28-day period covers only the brooding stage, leaving the growth trajectories during the rearing phase unexamined. Furthermore, this experiment did not employ routine immunization procedures (such as vaccination) to ensure the uniqueness of experimental variables. However, the immune system and gut development have complex interactions, and vaccination may affect villus morphology and crypt turnover by inducing local immune responses. Therefore, the absence of immunization procedures may cause some deviation between the results of this study and actual poultry production environments. Furthermore, this study primarily describes intestinal differences morphologically without exploring molecular mechanisms. Research suggests that epithelial cell proliferation is regulated by signalling pathways such as Wnt/β-catenin and Notch [45], while the GH-IGF-1 axis plays a key role in mediating growth differences [46]. Additionally, early gut microbiota colonisation profoundly influences morphological development [47]. Future research could integrate transcriptomics and 16S rRNA sequencing to further resolve the molecular genetic mechanisms and microecological foundations underlying these developmental disparities.

## 5. Conclusions

This study confirms that AA broilers establish a foundation for high-efficiency nutrient conversion through an intestinal morphology characterised by high villus height, intestinal density, and V/C ratios. In contrast, while YYJ compensates for absorptive deficiencies by increasing intestinal length and weight, the limitations in unit efficiency significantly restrict its growth rate, revealing that the relatively slower development of intestinal mucosal architecture in the early stage is one of the important physiological constraints associated with the lower growth potential of indigenous breeds compared to modern commercial lines. These results provide a scientific basis for the germplasm improvement of YYJ. Directed optimisation of early intestinal development, while maintaining stress resistance and flavour, represents a critical strategy for overcoming growth bottlenecks in this breed.

## Figures and Tables

**Table 1:** Comparison of hatching egg weight and body weight at different ages between YYJ and AA broilers.

Indicators	YYJ	AA Broilers
Egg weight (g)	51.35 ± 3.27 **	62.87 ± 3.34 **
1-day weight (g)	34.64 ± 2.77 **	44.71 ± 3.57 **
14-day weight (g)	161.38 ± 10.86 **	433.96 ± 41.79 **
28-day weight (g)	394.49 ± 42.77 **	1279.56 ± 169.87 **

Note: ** indicates a significantly correlation (*p* < 0.01); YYJ denotes Liangshan Yanying chicken; AA broilers denotes Arbor Acres chicken. Data are presented as mean ± SD.

**Table 2:** Comparison of growth performance between YYJ chickens and AA broilers.

Indicators	1–14 Days Old		15–28 Days Old		1–28 Days Old	
	YYJ	AA Broilers	YYJ	AA Broilers	YYJ	AA Broilers
ADG (g/d)	9.10 ± 0.94 **	26.13 ± 1.90 **	16.65 ± 2.50 **	53.49 ± 5.82 **	12.85 ± 1.50 **	39.92 ± 3.38 **
ADFI (g/d)	16.88 ± 1.17 **	28.92 ± 0.93 **	36.14 ± 1.73 **	90.56 ± 1.26 **	25.47 ± 0.74 **	60.16 ± 1.47 **
FCR	1.72 ± 0.17 **	1.07 ± 0.08 **	2.07 ± 0.26 **	1.68 ± 0.22 **	1.95 ± 0.20 **	1.48 ± 0.15 **

Note: ** indicates a significantly correlation (*p* < 0.01); YYJ denotes Liangshan Yanying chicken; AA broilers denotes Arbor Acres chicken. Data are presented as mean ± SD.

**Table 3:** Correlation coefficient matrix of egg weight and body weight at different ages for YYJ and AA broilers.

Indicators	Egg Weight (g)	1-Day Weight (g)	14-Day Weight (g)	28-Day Weight (g)
Egg weight (g)	1.000	0.620 **	0.405 *	0.331
1-day weight (g)	0.823 **	1.000	0.416 *	0.343
14-day weight (g)	0.496 *	0.552 **	1.000	0.495 *
28-day weight (g)	0.268	0.393	0.490 *	1.000

Note: The upper and lower triangles represent the correlation and significance tests between various indicators for YYJ and AA broilers, respectively. ** indicates a significantly correlation (*p* < 0.01); * indicates a significant correlation (*p* < 0.05). YYJ denotes Liangshan Yanying chicken; AA broilers denotes Arbor Acres chicken. Data are presented as mean ± SD.

**Table 4:** Comparison of relative intestinal length, relative weight, and density between YYJ and AA broilers.

Indicators	1 Day Old		14 Days Old		28 Days Old	
	YYJ	AA Broilers	YYJ	AA Broilers	YYJ	AA Broilers
Relative Length RL (%)						
Duodenum	26.94 ± 3.46 **	24.34 ± 2.57 **	8.53 ± 0.22 **	5.45 ± 0.33 **	4.58 ± 0.49 **	2.20 ± 0.24 **
Jejunum	44.09 ± 6.47 *	47.92 ± 5.54 *	15.44 ± 1.63 **	11.39 ± 0.58 **	8.54 ± 1.02 **	4.50 ± 0.47 **
Ileum	43.19 ± 5.94	45.77 ± 3.75	15.97 ± 0.78 **	10.78 ± 0.76 **	8.81 ± 1.30 **	4.91 ± 0.54 **
Relative Weight RW (%)						
Duodenum	0.80 ± 0.18 **	0.98 ± 0.15 **	0.59 ± 0.09 **	0.68 ± 0.06 **	0.54 ± 0.15	0.47 ± 0.09
Jejunum	0.97 ± 0.28 **	1.35 ± 0.19 **	0.90 ± 0.08 **	1.36 ± 0.25 **	1.04 ± 0.15 **	0.84 ± 0.10 **
Ileum	0.90 ± 0.27 **	1.43 ± 0.19 **	0.88 ± 0.05 **	1.02 ± 0.12 **	0.82 ± 0.13 **	0.67 ± 0.04 **
Density ID (g/cm)						
Duodenum	0.03 ± 0.00 **	0.04 ± 0.01 **	0.07 ± 0.01 **	0.12 ± 0.01 **	0.12 ± 0.02 **	0.21 ± 0.03 **
Jejunum	0.02 ± 0.00 **	0.03 ± 0.00 **	0.06 ± 0.00 **	0.12 ± 0.02 **	0.12 ± 0.02 **	0.19 ± 0.02 **
Ileum	0.02 ± 0.00 **	0.03 ± 0.00 **	0.06 ± 0.00 **	0.09 ± 0.01 **	0.09 ± 0.01 **	0.14 ± 0.01 **

Note: ** indicates a significantly correlation (*p* < 0.01); * indicates a significant correlation (*p* < 0.05), YYJ denotes Liangshan Yanying chicken; AA broilers denotes Arbor Acres chicken. Data are presented as mean ± SD.

**Table 5:** Comparison of intestinal villus height, crypt depth, and V/C ratio between YYJ and AA broilers.

Indicators	1 Day Old		14 Days Old		28 Days Old	
	YYJ	AA Broilers	YYJ	AA Broilers	YYJ	AA Broilers
VH (µm)						
Duodenum	236.08 ± 27.73 *	270.16 ± 53.12 *	402.65 ± 76.96 **	619.13 ± 84.55 **	515.98 ± 51.26 **	920.27 ± 39.41 **
Jejunum	198.17 ± 28.17	211.89 ± 42.25	481.31 ± 30.42 **	648.08 ± 79.80 **	473.94 ± 66.71 **	905.42 ± 72.38 **
Ileum	182.67 ± 15.58 **	254.31 ± 44.74 **	427.68 ± 80.13 **	565.12 ± 83.73 **	478.94 ± 92.86 **	911.13 ± 85.33 **
CD (µm)						
Duodenum	89.43 ± 15.64	90.67 ± 17.73	110.40 ± 19.87 **	143.43 ± 28.37 **	126.91 ± 31.59	120.55 ± 23.06
Jejunum	67.45 ± 12.81 **	89.30 ± 24.20 **	92.21 ± 16.74 **	118.69 ± 20.94 **	86.31 ± 22.82 **	135.77 ± 20.03 **
Ileum	79.21 ± 17.14	85.74 ± 22.07	94.44 ± 20.29 **	133.25 ± 20.80 **	101.53 ± 31.19	114.35 ± 24.42
V/C						
Duodenum	2.71 ± 0.56	3.09 ± 0.95	3.37 ± 1.08 **	5.27 ± 1.00 **	4.84 ± 1.16 **	6.65 ± 1.31 **
Jejunum	3.02 ± 0.65 *	2.47 ± 0.65 *	5.95 ± 1.63	4.91 ± 1.12	5.31 ± 1.29 **	7.89 ± 1.70 **
Ileum	2.41 ± 0.57 *	3.26 ± 1.36 *	4.39 ± 0.81	5.13 ± 1.28	5.27 ± 1.43 **	7.03 ± 1.50 **

Note: ** indicates a significantly correlation (*p* < 0.01); * indicates a significant correlation (*p* < 0.05). VH denotes villus height, CD denotes crypt depth, and V/C denotes the villus-crypt ratio. YYJ denotes Liangshan Yanying chicken; AA broilers denotes Arbor Acres chicken. Data are presented as mean ± SD.

## Data Availability

The raw data supporting the conclusions of this article will be made available by the authors on request.

## Supplementary Materials

Supplementary Materials: The following supporting information can be downloaded at: https://www.mdpi.com/article/10.3390/ani16060991/s1, Table S1: Guaranteed values of feed composition analysis for YYJ and AA broiler.
