# Supplementary material for: Comparative Growth Performance and Intestinal Morphological Development Between Liangshan Yanying Chicken and Arbor Acres Chicken During the Brooding Stage
Source: Animals (Basel). 2026 Mar 22;16(6):991. doi: 10.3390/ani16060991 (PMC13024256; doi:10.3390/ani16060991)
Supplement: Supplementary file 1 [file animals-16-00991-s001.zip › animals-4193508-supplementary.pdf]

Table S1. Guaranteed values of feed composition analysis for YYJ and AA broiler

**composition**	**content**
Moisture（%）	≤14.0
Crude protein（%）	≥21.0
Crude fiber（%）	≤5.0
Crude ash（%）	≤8.0
Calcium（%）	0.8~1.3
Total phosphorus（%）	≥0.6
Sodium chloride（%）	0.3~0.8
Methionine（%）	0.5~0.9
